# Electroacupuncture modulates abnormal brain connectivity after ischemia reperfusion injury in rats: A graph theory‐based approach

**DOI:** 10.1002/brb3.3504

**Published:** 2024-05-02

**Authors:** Si‐Si Li, Xiang‐Xin Xing, Xu‐Yun Hua, Yu‐Wen Zhang, Jia‐Jia Wu, Chun‐Lei Shan, He Wang, Mou‐Xiong Zheng, Jian‐Guang Xu

**Affiliations:** ^1^ School of Rehabilitation Science Shanghai University of Traditional Chinese Medicine Shanghai China; ^2^ Department of Physical Medicine and Rehabilitation The Second Affiliated Hospital and Yuying Children's Hospital of Wenzhou Medical University Wenzhou China; ^3^ Center of Rehabilitation Medicine Yueyang Hospital of Integrated Traditional Chinese and Western Medicine Shanghai University of Traditional Chinese Medicine Shanghai China; ^4^ Department of Traumatology and Orthopedics Yueyang Hospital of Integrated Traditional Chinese and Western Medicine Shanghai University of Traditional Chinese Medicine Shanghai China; ^5^ Institute of Science and Technology for Brain‐Inspired Intelligence Fudan University Shanghai China; ^6^ Engineering Research Center of Traditional Chinese Medicine Intelligent Rehabilitation Ministry of Education Shanghai China

**Keywords:** betweenness centrality, degree centrality, electroacupuncture, ischemia reperfusion, topological property

## Abstract

**Background:**

Electroacupuncture (EA) has been shown to facilitate brain plasticity‐related functional recovery following ischemic stroke. The functional magnetic resonance imaging technique can be used to determine the range and mode of brain activation. After stroke, EA has been shown to alter brain connectivity, whereas EA's effect on brain network topology properties remains unclear. An evaluation of EA's effects on global and nodal topological properties in rats with ischemia reperfusion was conducted in this study.

**Methods and results:**

There were three groups of adult male Sprague‐Dawley rats: sham‐operated group (sham group), middle cerebral artery occlusion/reperfusion (MCAO/R) group, and MCAO/R plus EA (MCAO/R + EA) group. The differences in global and nodal topological properties, including shortest path length, global efficiency, local efficiency, small‐worldness index, betweenness centrality (BC), and degree centrality (DC) were estimated. Graphical network analyses revealed that, as compared with the sham group, the MCAO/R group demonstrated a decrease in BC value in the right ventral hippocampus and increased BC in the right substantia nigra, accompanied by increased DC in the left nucleus accumbens shell (AcbSh). The BC was increased in the right hippocampus ventral and decreased in the right substantia nigra after EA intervention, and MCAO/R + EA resulted in a decreased DC in left AcbSh compared to MCAO/R.

**Conclusion:**

The results of this study provide a potential basis for EA to promote cognitive and motor function recovery after ischemic stroke.

## Graphical abstract

Graph theory studies of functional networks show significant changes in the brain regions associated with the process of cognitive and motor functions after ischemic stroke. Electroacupuncture at LI11 and ST36 acupoints could affect the network topological measures in cognitive and motor‐related brain areas, and these changes are related to acupoint specificity.

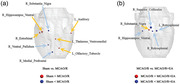

## 1 INTRODUCTION

Stroke is one of the most common, disabling, and potentially deadly pathologies worldwide, and ischemic stroke represents over 80% of all cases (Chen et al., 2017). Ischemic strokes caused by cerebral artery occlusion can lead to impairments in motor, sensory, or cognitive function (Heo et al., 2020; Li, Hua, et al., 2021). According to an increasing number of studies, enhancing brain plasticity has an important role in functional recovery from dysfunction in cerebral infarction (Choi et al., 2019; Zhang et al., 2019). Widely distributed brain networks are strongly associated with functional recovery after acute ischemic stroke (Cheng et al., 2016). The brain regions surrounding or connected to the infarcted lesion may undergo structural and functional changes as a result of stroke, understanding the influence of cerebral ischemia‒reperfusion injury on the brain network may help guide rehabilitation strategies for stroke patients (Broome et al., 2019; Toscano et al., 2019).

The brain is a complex network composed of highly interconnected regions with functional connections between adjacent and distant brain areas (Aihara et al., 2020; Coelho et al., 2021). Focal cerebral infarctions can have catastrophic and unexpected consequences in the whole‐brain network. Resting state functional magnetic resonance imaging (rs‐fMRI) has become a noninvasive and effective tool to explore brain function (Xu, 2015). Graph theoretical approaches have recently been proven to be a powerful framework for assessing functional connectivity of fMRI brain networks (Coloigner et al., 2017). Graph theory emerged as a method of network analysis and viewed the brain as a complex functional system exhibiting topological characteristics, including nodal centrality and small‐world properties (Sporns, 2018). The small‐world model can reflect the integration and separation of information transfer and is therefore well suited to investigate complex brain networks (Liao et al., 2017). Disrupted whole‐brain network topology is likely to develop in individuals with stroke (Chen et al., 2020). Based on graph theoretical approaches, the findings of Vecchio et al. (2023) demonstrated that acute stroke altered global functional connectivity as well as the balance between network segregation and integration. According to Pang et al. (2022), the clustering coefficient and transitivity of stroke patients were higher, the global efficiency and small‐worldness being smaller than those of healthy individuals; besides, the clustering coefficients and local efficiency negatively correlate with motor function in patients. Zhu et al. (2017) reported that small‐world network organization was observed in both acute ischemic stroke and healthy control (HC) groups, and acute ischemic stroke patients had a shorter shortest path length and higher global efficiency compared with HC, suggesting a tendency for functional networks to randomize in patients. In another study, it was stated that a shift toward a regular configuration of brain networks was observed in the functional connectome of unilateral acute brainstem ischemic stroke patients (Shi et al., 2021). The reorganization of the entire brain pattern during stroke recovery is still disputable and elusive for now.

Acupuncture, an important intervention in traditional Chinese medicine (TCM), has been extensively used to treat human disease for over 2000 years in China (Lai et al., 2020; Yang et al., 2022). Previous studies on the acupuncture mechanism suggest that acupuncture can be used to adjust the balance of qi and blood in the human body and then enhance human body functions by stimulating the acupoints (Han, Cui, et al., 2020; Urits et al., 2020). Electroacupuncture (EA) is a modern acupuncture therapy that involves electrical stimulation of acupuncture points to enhance efficacy (Guo et al., 2023). Numerous research studies have indicated that acupuncture can regulate resting‐state brain activity or connectivity (Cai et al., 2018; Li, Cai, et al., 2021). Acupuncture at acupoints has been shown to specifically activate functional activities in certain brain regions (Liu et al., 2015). There are two acupoints that are commonly used in Chinese clinics for stroke treatment: Quchi (LI11) and Zusanli (ST36) (Huang et al., 2017). The previous brain imaging research has proven that the functional whole‐brain network architecture of healthy individuals is altered after EA intervention, which confirms the brain function related to the specificity of meridians and acupoints (Han, Jin, et al., 2020). A meta‐analysis study with ST36 showed that it was able to activate the left cerebellum, the bilateral Rolandic operculum, the right supramarginal gyrus, and the right cerebellum, and acupuncture at ST36 was primarily associated with perception and action according to functional characterizations (Zhang et al., 2023).

The previous study identified that stroke significantly reduces the small‐worldness and the global and mean local efficiency of the whole‐brain network in patients, and acupuncture at GB34 could instantly increase the clustering coefficient and the mean local efficiency of the whole‐brain network, aiding in the reorganization of the disrupted poststroke whole‐brain network (Han, Jin, et al., 2020). Another study focused on the effects of EA on global efficiency and characteristic path length, and the results showed that the global efficiency was significantly increased and the characteristic path length was significantly decreased after EA at ST36 and LI11 compared to the middle cerebral artery occlusion (MCAO) group (Yin et al., 2022). However, less is known about the effect of EA on topological properties of the networks following ischemia stroke. Hence, we used graph theory methods and topological indices to explore the mechanism of EA.

Therefore, MCAO/reperfusion (MCAO/R) model was used in the current study, and we performed rs‐fMRI with graph theory analysis to detect and examine the topological organization of functional networks within the brain in rats receiving MCAO/R injury, and EA's function was investigated.

## 2 MATERIALS AND METHODS

### 2.1 Animals

The Shanghai Laboratory Animal Research Center (Shanghai, China) provided us with 24 Sprague‐Dawley inbred male rats weighing 250–280 g, of clean grade. There were eight animals per group to obtain for a valid statistical outcome. The rats were kept under temperature‐controlled environment at 23 ± 2°C with a cycle of 12‐h light/12‐h dark. Rats were given with standard food pellets and bottled water ad libitum. The Committee on Animal Care and Usage of Shanghai University of Traditional Chinese Medicine approved all experimental procedures using animals (approval No. PZSHUTCM200110002). Research with laboratory animals is handled by following the National Institutes of Health Guidelines for the Care and Use of Laboratory Animals.

### 2.2 Focal cerebral ischemia reperfusion model

According to the previous description, the MCAO/R model was developed (Longa et al., 1989). In short, we anesthetized the rats by injecting sodium pentobarbital (30 mg/kg) intraperitoneally. We carefully isolated the left external carotid artery (ECA), the internal carotid artery (ICA), and the common carotid artery. ECA was incised with a small incision after ligated at the distal end of the ECA. Subsequently, it was gently inserted from the ECA stump with a monofilament nylon suture purchased from Guangzhou Jialing Biotechnology Co., Ltd and then carefully introduced into the ICA lumen to occlude the MCA. Reperfusion was achieved after 2 h of ischemia by slowly withdrawing the nylon monofilament and restoring the brain blood flow of the ischemic area. Rats were kept warm with a heating pad during the experiments. Use a baking lamp to irradiate the animals before they wake up from anesthesia after surgery to maintain their body temperature. The rats were weak after operation, put the rat feed and water in a bowl, and then put the bowl in the cage to facilitate the rats to drink and eat. When the rat appeared extreme emaciation, the rat underwent euthanasia as the humanitarian end point of the study. We randomly divided rats into sham group, MCAO/R group, and MCAO/R + EA group. Eight rats per group were used. The same operation was conducted in the sham group without the monofilament inserted.

### 2.3 EA intervention

**FIGURE 1:**
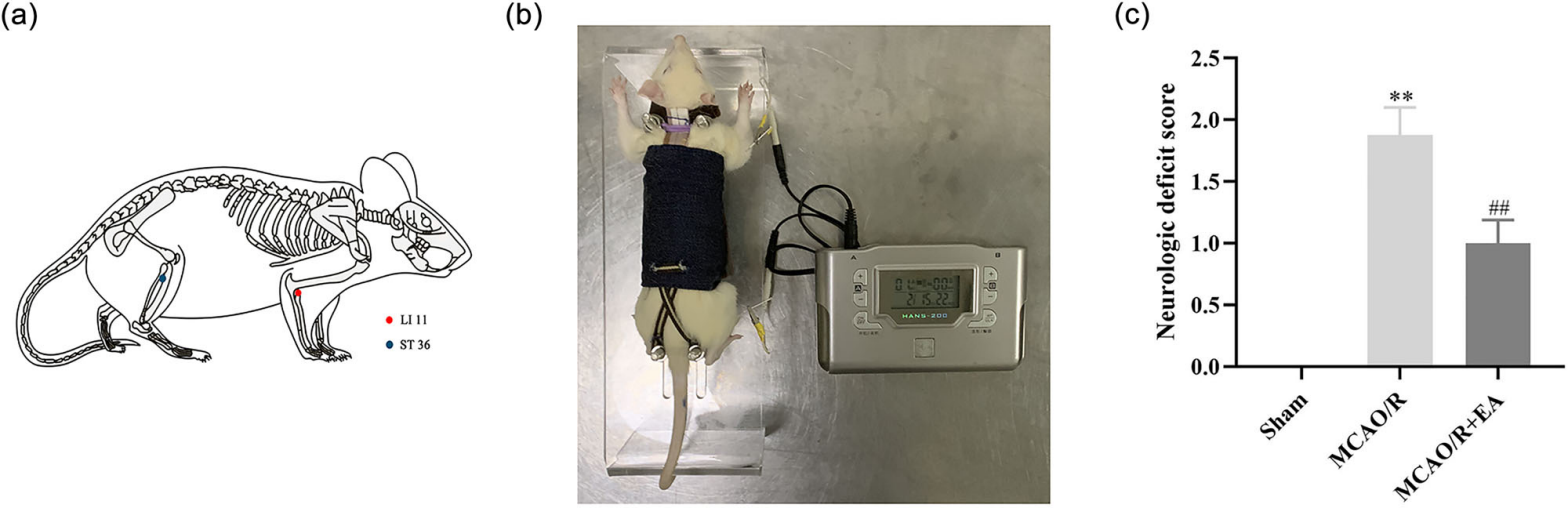
Electroacupuncture (EA) intervention improved the neurological deficit 7 days after middle cerebral artery occlusion/reperfusion (MCAO/R). (A) The locations of acupoints. (B) Rats are treated with EA while awake. (C) Neurologic deficit score of rats at 7 days after MCAO/R. The results are expressed as ±SEM (*n* = 8). ^**^ *p* < .01 compared with the sham group, ^##^ *p* < .01 compared with the MCAO/R group.

The EA intervention was continued for 7 days, once daily at the same time (9:00 a.m.). The rats were gently immobilized in a fixation apparatus appropriate to their size, though they could still freely move their head and limbs. To minimize the stress of immobilization, rats were allowed to acclimate to the fixation apparatus at least 3 days before the EA intervention. EA was performed on the right limbs at LI11 and ST36 acupoints using needles (0.25 × 13 mm). As shown in Figure 1, LI11 was located in the anterolateral depression of the radial joint, and ST36 was located 5 mm below the capitulum fibulae (Liu et al., 2016). The device used was the HANS‐200E stimulator (Nanjing Jisheng Co.) for EA intervention with a frequency of 2/15 Hz and 0.1 mA intensity.

### 2.4 Neurologic deficit scores

Neurologic deficit scores were obtained 7 days after EA invention based on Zea Longa scores (Longa et al., 1989). The scoring system was defined as follows: 0, no deficit; 1, unable to stretch the contralateral forelimb fully; 2, circling to the contralateral side; 3, falling over to the contralateral side; and 4, did not walk spontaneously.

### 2.5 fMRI data acquisition and preprocessing

The rs‐fMRI data were obtained after 7 days of EA intervention with an 11.7 T small animal imaging system (Bruker Corporation). A 5% isoflurane anesthesia was administered to the rat and then fixed the rat in the scanner. Continuous anesthesia (1.5% isoflurane + 0.05 mg/kg dexmedetomidine) was administered during scanning, and respiration was monitored by a ventilator. In order to obtain the rs‐fMRI data, echo planar imaging was conducted, and the parameters were as follows: The number of time point is 200, data matrix = 64 × 64, field of vision = 27 × 27 mm^2^, repetition time = 3000 ms, echo time = 8.142 ms, flip angle = 90°, slice thickness = 0.3 mm, and number of averages = 1. The total time of scanning was approximately 25 min for each group. Images were checked immediately after data collection.

We applied the MATLAB 2013b platform (MathWorks, Inc.) with the SPM12 toolbox (http://www.fil.ion.ucl.ac.uk/spm/) to handle rs‐fMRI data. A conversion of Bruker images to NIFTI was performed, and functional images from the first 10 volumes were deleted due to MRI signal instability. Then, the images were processed with slice timing correction, coregistration, and realignment for head motion correction using MRIcron software (www.mricro.com), and nonbrain tissue was removed from the images. Furthermore, the orientation of all images was modified according to a standard template (Schwarz et al., 2006) through adjust pitch/roll/yaw parameters. Subsequently, the images were normalized and resampled to 2.06 × 2.06 × 2 mm^3^. After normalization, smoothing was performed using a full width at half maximum triploid as the voxel size (6.18 × 6.18 × 6 mm^3^) to enhance the ratio of signal/noise in the images. Further preprocessing included temporal detrending and filtering (0.01−0.1 Hz), and the covariates were regressed out, including white matter signals, cerebrospinal fluid signals, and head movement signals.

### 2.6 Brain network construction and analysis

In order to construct the functional brain network, the Graph‐theoretical Network Analysis Toolkit (GRETNA, www.nitrc.org) was used. The entire brain was divided into 96 anatomical regions in line with the Schwarz's standard space (Schwarz et al., 2006). In addition, the average time series were extracted for each of the 96 MNI regions. Then, the relation matrix was converted into a binary connection matrix by the selected threshold of the *z* value, and a graphic model of the functional network was constructed. The range of sparsity was set from 0.05 to 0.5 with an interval of 0.01, as recommended by a prior study. GRETNA was employed for the calculation of network topology properties. We calculated the network measurements of shortest path length (*Lp*), global efficiency (*E_glob_*), local efficiency (*E_loc_*), and small‐worldness index. Betweenness centrality (BC) and degree centrality (DC) were also used to illustrate the key nodes in functional networks.

*The shortest path length*: The shortest path length takes the mean of the length of the shortest path between nodes *i* and *j* (Tao et al., 2015).

In the network, *G* is defined in the following formula:

$$Lp\left(G\right)=\frac{1}{N\left(N-1\right)}\sum_{i\neq j\in N}L_{ij}$$

where *L_ij_* is the length of the shortest path between node *i* and node *j*.

*Global efficiency*: Global efficiency refers to the information transmission efficiency, which reflects the information transmission capacity of the whole brain network (Latora & Marchiori, 2001).

In the network, *G* is defined in the following formula:

$$E_{glob}\left(G\right)=\frac{1}{N\left(N-1\right)}\sum_{i\neq j\in G}\frac{1}{L_{ij}}$$

*Local efficiency*: Local efficiency refers to efficiency computed on node neighborhoods (Ma et al., 2017).

In the network, *G* is defined in the following formula:

$$E_{loc}\left(G\right)=\frac{1}{N}\sum_{i\in G}E_{glob}\left(G_{i}\right)$$

where *G_i_* is the subgraph which comprises the direct neighbors of node *i*.

*Small world*: Small‐worldness index means the ratio of coefficient (*Cp*) and *Lp*. The network is considered to have the small‐world property when Sigma > 1 (Watson et al., 2018):

$$small-worldness\;index=\frac{Cp/Cp_{rand}}{Lp/Lp_{rand}}$$

where *Cp_rand_* and *Lp_rand_* represented the mean value of *Cp* and *Lp* of 100 random networks.

*Degree*: The DC is a graph theory metric that measures how many edges connected to a node and reflects the node's ability to connect directly with other nodes, having a neurobiological basis (Kwon et al., 2019).

In a network, *G* with *N* nodes and *K* edges, the formula is defined as

$$k\left(i\right)=\sum_{i\neq j\in G}a_{ij}$$

where *a_ij_* is the link between node *i* and node *j*.

*Betweenness centrality*: Betweenness centrality describes the proportion of shortest paths that pass through a node in a network (Marzullo et al., 2019).

Betweenness centrality of node *i*,

$$BC\left(i\right)=\sum_{i\neq j\neq k\in G}\frac{L_{jk}\left(i\right)}{L_{jk}}$$

where *L_jk_* represents the length of shortest path between node *j* and node *k*, and *L_jk_* (*i*) represents the amount of shortest paths that pass through node *i*.

### 2.7 Statistical analysis

The condition *p* < .05 (uncorrected) in GRETNA software was set as the significance level for the network properties between groups. We calculated the network properties of groups at each selected threshold sparsity (0.05:0.01:0.5) (Chen et al., 2018; Xue et al., 2022), and the area under the curve (AUC) for each network property of each rat across all levels of sparsity was utilized for further statistical analysis. By one‐way analysis of variance (ANOVA) with least significant difference (LSD), AUCs were compared between three groups (sham, MCAO/R, and MCAO/R + EA), using SPSS (IBM, SPSS Statistics). ANOVA was used for comparison neurologic deficit scores between multiple groups, and LSD was used for post hoc test. It was considered significant when the *p* value was less than .05.

## 3 RESULTS

### 3.1 EA intervention improved the neurological deficit in MCAO/R rats

As shown in Figure 1C, at 7 days after MCAO/R, higher neurologic deficit score was found in the MCAO/R group compared with the sham group (*p* < .01). However, a significantly lower neurological deficit score was observed in the MCAO/R + EA group compared to MCAO/R (*p* < .01). Thus, the EA intervention resulted in an improvement in neurologic function in MCAO/R rats.

### 3.2 Global topological properties

**FIGURE 2:**
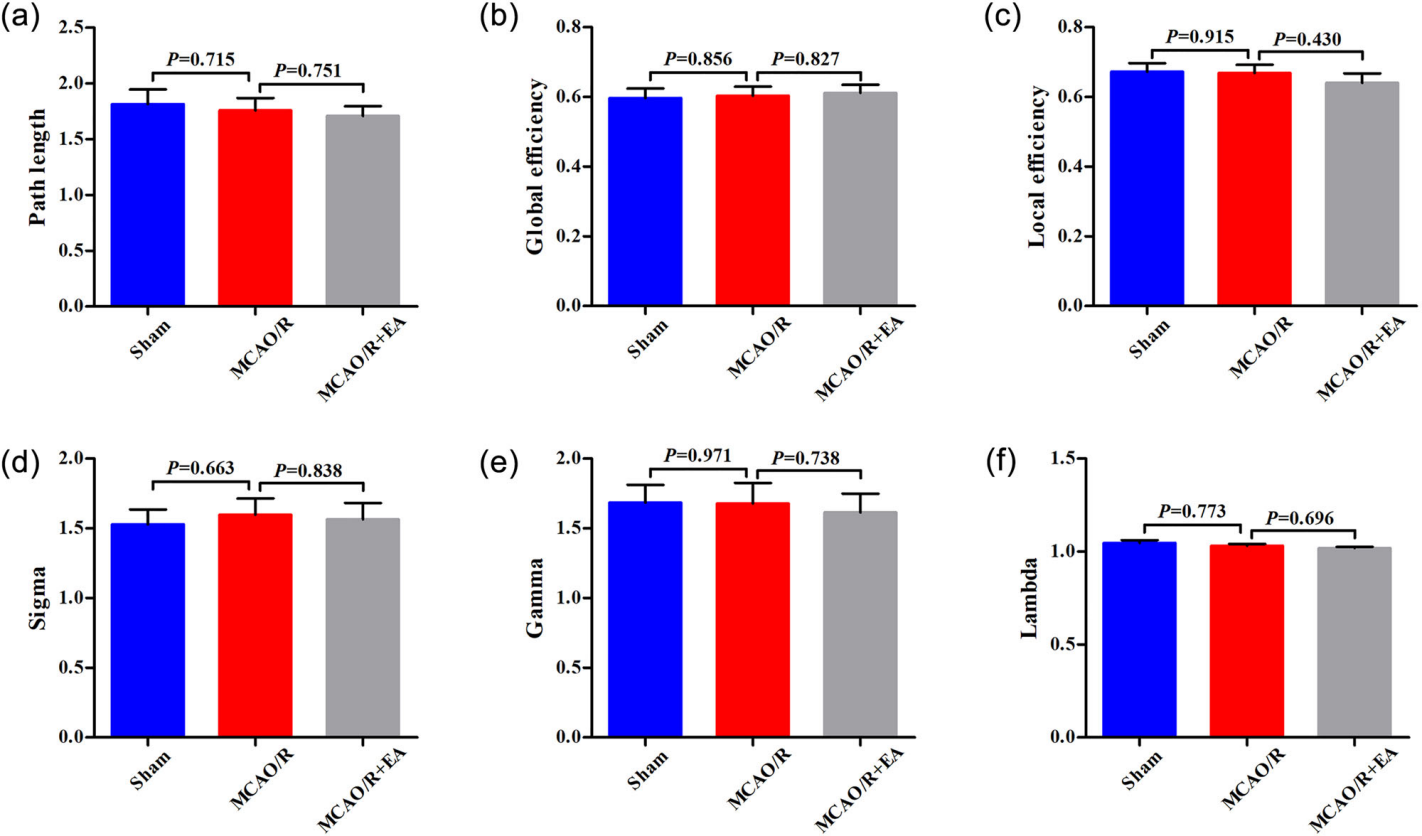
Global properties of the sham, middle cerebral artery occlusion/reperfusion (MCAO/R), and MCAO/R + electroacupuncture (EA) groups. (A) Path length. (B) Global efficiency. (C) Local efficiency. (D) Sigma. (E) Gamma. (F) Lambda. Bar graphs show the global parameters, with blue bars representing the sham group, red bars representing the MCAO/R group, and gray bars representing the MCAO/R + EA group.

**FIGURE 3:**
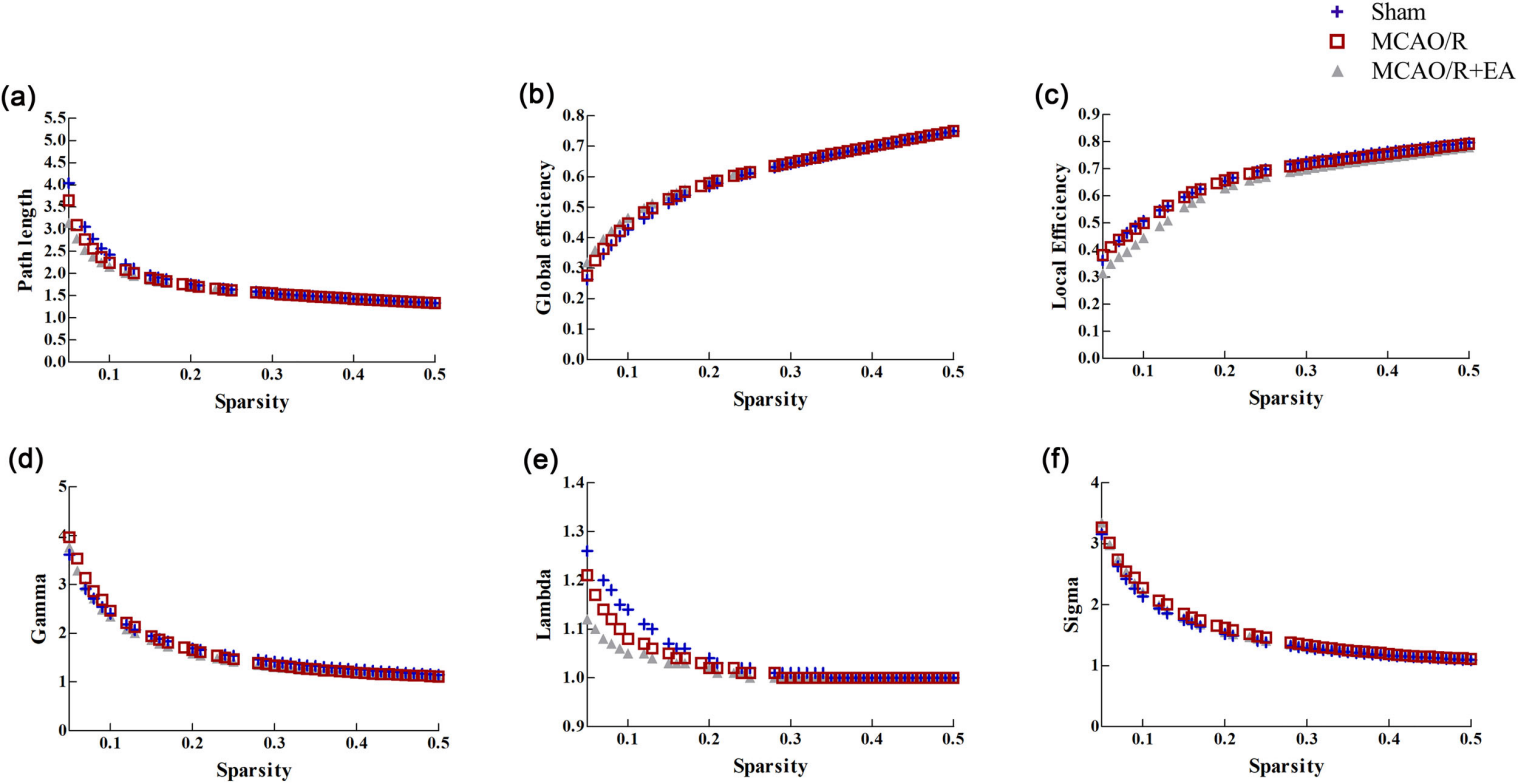
The global network properties of rats were computed over the sparsity range (0.05–0.5) with an interval of 0.01. (A) Path length. (B) Global efficiency. (C) Local efficiency. (D) Gamma. (E) Lambda. (F) Sigma. EA, electroacupuncture; MCAO/R, middle cerebral artery occlusion and reperfusion.

A comparison of the global measures between the sham group and MCAO/R group or between the MCAO/R group and MCAO/R + EA group is shown in Figures 2 and 3. Global properties were calculated at each sparsity threshold of the three groups in Figure 2. Figure 3 demonstrates the statistical results of the AUC for each network property of each rat across all levels of sparsity. No significant difference was noted between the sham group and MCAO/R group or between the MCAO/R group and MCAO/R + EA group in either *Lp*, *E_glob_*, or *E_loc_* under any of the selected spasticity thresholds (*p* > .05). Sigma represents small‐worldness, lambda represents clustering coefficient, and gamma represents characteristic path length, respectively. Small‐world properties can be measured using the ratio of gamma to lambda (i.e., sigma = gamma/lambda), with sigma values greater than 1 being considered small‐world. For the selected range of sparsity thresholds, rats in both groups all represented sigma >1, indicating rats still possessed small‐world property. However, no significant difference was found regarding the small‐worldness between groups (*p* > .05).

### 3.3 Nodal topological properties

**FIGURE 4:**
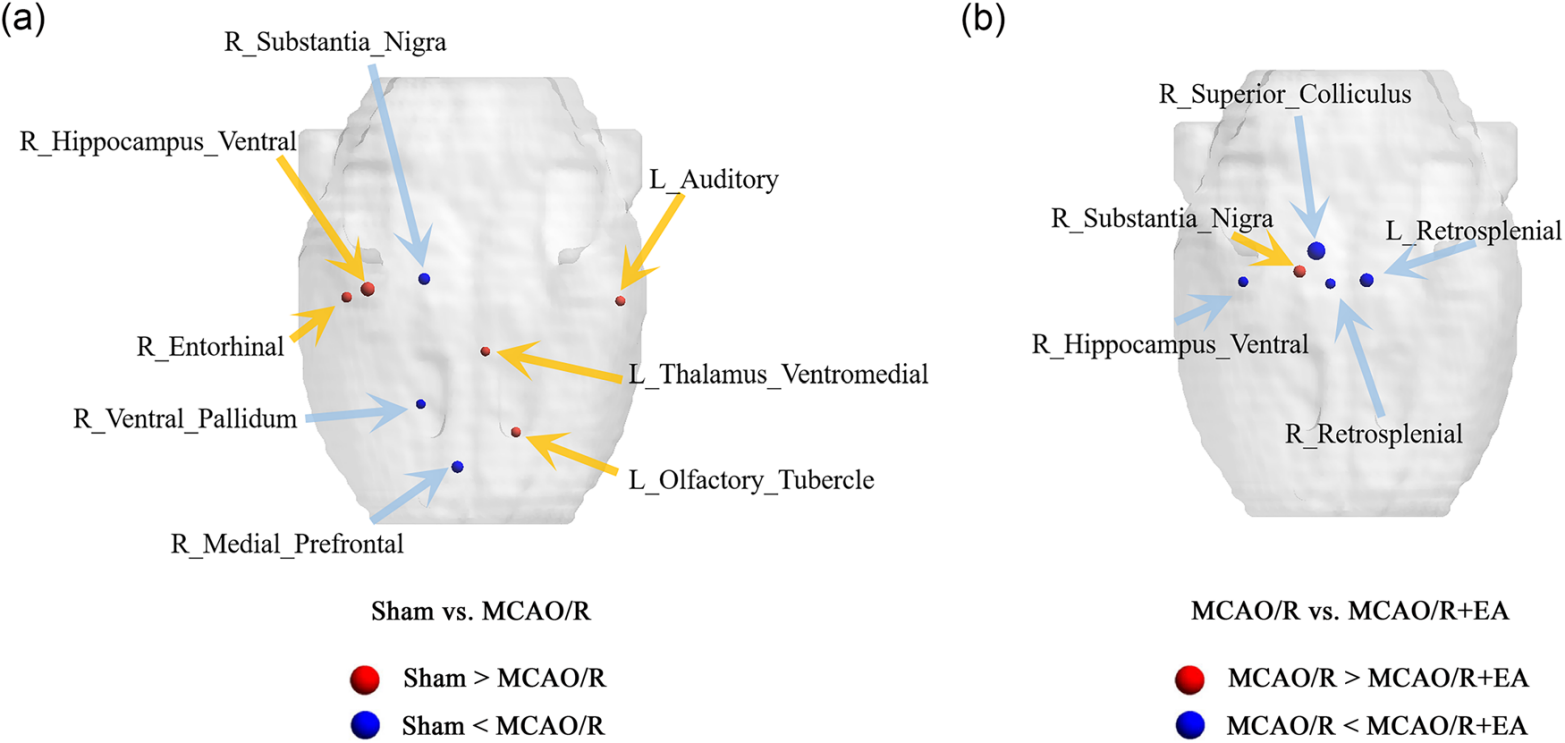
A comparison of betweenness centrality (BC) among groups. *p* < .05, uncorrected was used as the statistical criterion. (A) When sham versus middle cerebral artery occlusion/reperfusion (MCAO/R), the red ball indicates that the BC value of the sham group is enhanced, whereas the blue ball indicates that the BC value of the sham group is decreased. (B) When MCAO/R versus MCAO/R + electroacupuncture (EA), the red ball indicates that the BC value of the MCAO/R group is enhanced, whereas the blue ball indicates that the BC value of the MCAO/R group is decreased. The size of the ball represents *T*‐value, and larger ball‐size indicated higher *T*‐value.

**FIGURE 5:**
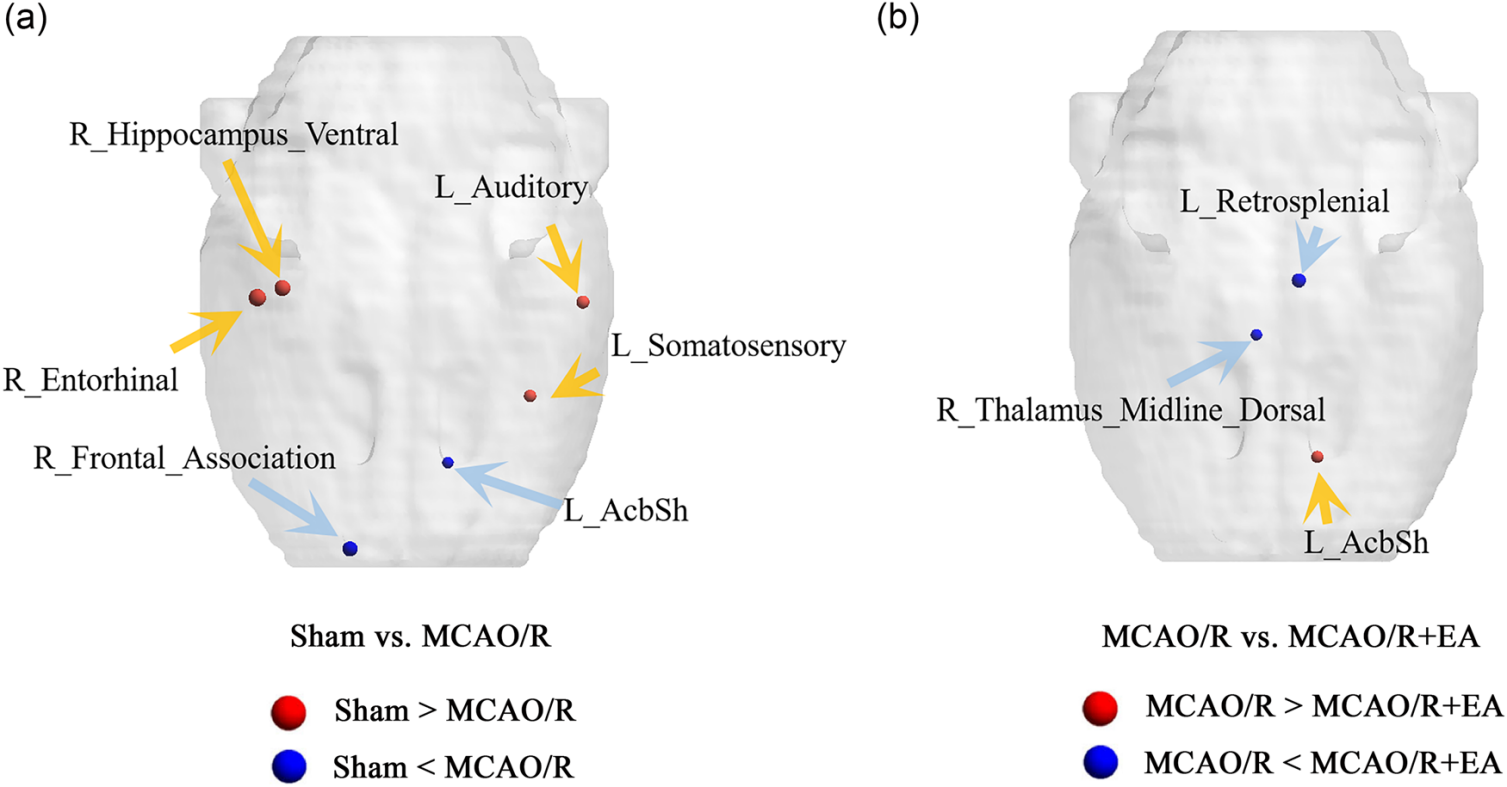
A comparison of degree centrality (DC) among groups. *p* < .05, uncorrected was used as the statistical criterion. (A) When sham versus middle cerebral artery occlusion and reperfusion (MCAO/R), the red ball indicates that the DC value of the sham group is enhanced, whereas the blue ball indicates that the DC value of the sham group is decreased. (B) When MCAO/R versus MCAO/R + electroacupuncture (EA), the red ball indicates that the DC value of the MCAO/R group is enhanced, whereas the blue ball indicates that the DC value of the MCAO/R group is decreased. The size of the ball represents *T*‐value, and larger ball‐size indicated higher *T*‐value. AcbSh, nucleus accumbens shell.

In our statistical analysis employing *t*‐tests, the findings were as follows: Table 1 presents the nodal metrics of brain areas that differed significantly between the three groups. In the MCAO/R group, BC values in the right ventral hippocampus were significantly lower than in the sham group, whereas BC values in the right substantia nigra were significantly higher than in the sham group. As compared to the MCAO/R group, the BC value was increased in the right hippocampus ventral and decreased in the right substantia nigra in the MCAO/R + EA group (Figure 4). The DC value in the left nucleus accumbens shell (AcbSh) of the MCAO/R group was higher compared with the sham group. There DC value in the left AcbSh in the MCAO/R + EA group was lower compared to the MCAO/R group (Figure 5). Statistical significance was defined as *p* < .05, uncorrected, for between‐group differences.

**TABLE 1:** The brain regions showing significant differences in nodal metric between the three groups.

Brain regions	Betweenness centrality		Degree centrality	
	*p* values	*T* values	*p* values	*T* values
**Sham > MCAO/R**				
R_Hippocampus_Ventral	0.005	3.259		
**Sham < MCAO/R**				
R_Substantia_Nigra	0.015	−2.755		
L_AcbSh	–	–	0.041	−2.245
**MCAO/R > MCAO/R + EA**				
L_AcbSh	–	–	0.034	2.353
R_Substantia_Nigra	0.012	2.875		
**MCAO/R < MCAO/R + EA**				
R_Hippocampus_Ventral	0.030	−2.407		

Abbreviations: AcbSh, nucleus accumbens shell; EA, electroacupuncture; MCAO/R, middle cerebral artery occlusion and reperfusion.

We have performed family‐wise error (FWE), false discovery rate (FDR), and AlphaSim correction; however, the results did not survive FDR, few, and AlphaSim correction. For values of BC and DC that showed significant differences in one‐way ANOVA, LSD post hoc test method was used to further compare the differences between groups. Only the left somatosensory cortex showed significant differences in DC.

## 4 DISCUSSION

The current study used graph theory analysis to detect the topological organization of the brain networks in rats with ischemic stroke treated by EA. The following results were obtained: (1) EA intervention improved the neurological deficit in MCAO/R rats. (2) No intergroup differences were found in global topological properties. (3) From the perspective of regional nodal characteristics, a significant increase in BC was found in the right ventral hippocampus following MCAO/R with EA administration, whereas the right substantia nigra showed a significant decrease in BC; in addition, the DC was lower in the left AcbSh compared with the MCAO/R group. This study shows EA's effect on the functional whole‐brain network after cerebral ischemia‒reperfusion injury.

EA is a historical and traditional treatment that has been available for a great variety of disorders for thousands of years. Previous studies suggest that EA at both low and high frequencies could induce a therapeutic effect on stroke. Han (2004) have indicated that EA stimulation with different frequencies can produce different biological effects, and low frequency stimulation can increase the release of various opioid peptides, including enkephalin, β‐endorphins, and endorphins, which acts on the μ and δ receptors of the central nervous system, whereas high frequency stimulation increases the release of dynorphin, which acts on the κ‐receptor of the spinal cord. Xiong et al. (2003) focused on the effects of three different frequencies of 2/5, 2/15, and 2/100 Hz on ischemic tolerance of brain tissue. The findings indicate that the repeated stimulation of acupoints with 2/15 Hz density wave can significantly improve ischemic tolerance of brain tissue. Dilating blood vessels surrounding ischemic penumbra is the primary treatment goal following focal cerebral ischemia in order to prevent or reverse brain damage (Chavez et al., 2017). Acupuncture is known to be an effective way to regulate blood flow. A study on cerebrovascular response after acupuncture in stroke patients found that acupuncture stimulation induced perifocal recombination by improving local cerebral blood flow (Lee et al., 2003). The study by Kim et al. (2018) showed that acupuncture improves cerebral vessel hemodynamic indices, especially the vertebral artery's blood flow quantity. According to another study conducted on 16 healthy volunteers suggested that EA might increase blood flow to the prefrontal cortex, reduce heart rate, and activate the parasympathetic nervous system via the trigeminovagal reflex by stimulating 1 cm from the front hairline midpoint and both sides at the inner edge of the eyebrows (Waki et al., 2017). Acupuncture can improve cerebral cortex function and enhance cerebral blood circulation by making blood vessels dilate and blood flow unobtrusive. The mechanism is primarily associated with the regulation role of acupuncture on nerves, immunity, and body fluids (Wang et al., 2019). A therapeutic principle of hemiplegia after stroke based on the theory of TCM is “Treating Flaccid Paralysis by Yangming Alone,” and the clinical application has always followed this principle (Zou et al., 2020). ST36 is considered the “sea” acupoint of the stomach meridian of Foot Yangming, LI11 is regarded as the “sea” acupoint of the large intestine meridian of Hand Yangming, and therefore, they are often prescribed as the interventional target of ischemic stroke, all located near to the joints of limbs; thus, they are contributed to the recovery of motor dysfunction following stroke. Liu et al. found that functional brain networks had the characteristics of small‐world networks under each condition, whereas the functional network had increased local efficiency upon acupuncture stimulation at ST36. In several regions of the limbic system, prefrontal lobe, parietal lobe, temporal lobe, and occipital cortex, the degree of nodes has been profoundly affected (Liu et al., 2012).

The plasticity of the adult brain after stroke depends on the presence of many diffuse and redundant connections in the central nervous system as well as the ability to develop new functional and structural circuits by remapping cortical regions that are related (Murphy & Corbett, 2009). However, little attention has been given to the brain's neuroplasticity in relation to acupuncture for stroke. In conjunction with fMRI technology, the central nervous effect of acupuncture on stroke could be fully explored anatomically as well as functionally. Cognitive and motor dysfunctions are the main clinical features after stroke (Lu et al., 2007). In a study using graph theory methods pointed out that the subcortical injury is limited to the right hemisphere motor pathway, acupuncture could help to reconstruct the damaged whole brain network after stroke (Han, Jin, et al., 2020). Further, acupuncture also affects cognitive function in addition to motor function after stroke (Zhang et al., 2021). This study analyzed the hub properties of nodes from a BC perspective, which is an indicator of a node's functional importance in terms of serving as a bridge for information processing. In general, information exchange and integration are both greatly facilitated by nodes with high BC (Wanni Arachchige et al., 2021). Wang et al. (2022) compared BC values between normal persons and ischemic moyamoya disease patients, a type of chronic occlusive cerebrovascular disease, and the results demonstrated that the BC values of the left medial superior frontal gyrus and left hippocampus were significantly reduced in the patients. The hippocampus is one of the brain structures more crucially involved in cognitive functions (Shetty et al., 2017). Previous study has shown that the function and structure of the hippocampus are significantly changed after stroke (Sanchez‐Bezanilla et al., 2020). BC value in the right ventral hippocampus in the MCAO/R group was significantly lower than in the sham group, and the BC value was increased in the right hippocampus after EA treatment in our study. A stroke event affected not only the lesioned hemisphere but also the contralesional hemisphere, which could be due to the complex interconnections and dependencies between brain regions (Zhang et al., 2020). Previous studies shed some light on this phenomenon from the perspectives of neuroimaging and neurobiology. It was found that not only the dendrites and dendritic spines of the ipsilateral hippocampus were damaged, but also the dendrites and dendritic spines of the contralateral hippocampus were damaged after acute stroke, which influenced the hippocampus to exert normal biological functions (Hu et al., 2020). A recent clinical study indicated a unilateral posterior stroke resulted in a contralateral hippocampal impairment and produced an object‐recognition memory impairment, which may be related to the cerebellum’ s direct and indirect reciprocal projections to various areas of the forebrain, including the hippocampus (Moreno et al., 2022). Another study calculated the ratios of *n*‐acetylaspartate/creatine (NAA/Cr) obtained by using software integrated in the MR scanner, and the results indicated that a reduced NAA/Cr ratio was also observed in the contralateral hippocampus in patients with unilateral MCAO when compared to controls, which means neuronal damage or dysfunction and is associated with cognitive impairment following a stroke (Tang et al., 2012). Furthermore, there are also studies showing that EA contributes to neuroplasticity and improves stroke recovery (Chavez et al., 2017; Li et al., 2022).

Additionally, the substantia nigra is an area of the midbrain that contains a widely studied group of dopamine cells, which mainly project to the striatum and are traditionally associated with the control center of motor activity (Fan et al., 2011; Lessard & Couture, 2001). In the process of cerebrovascular injury, in addition to causing infarction and hemorrhagic lesions, secondary degeneration may occur at the distance of the main vessels. This secondary degeneration includes the degeneration of the substantia nigra caused by infarction of the striatum (Ohe et al., 2013). Cranial MRI can be used to evaluate this secondary degeneration. As for the midbrain black quality degradation, Ohe et al. observed head MRI changes in a rat model of focal ischemia caused by MCA occlusion, and the results of their research indicated that the consumption of glucose and blood flow in the ipsilateral substantia nigra was increased after focal cerebral ischemia, and the occurrence of degeneration of neurons in the substantia nigra may be due to the transsynaptic, neurotransmitter mediated inhibition disinhibition associated with the destruction of the striatum (Nakane et al., 1997; Ohe et al., 2013). Consistent with our study, increased BC in the contralateral substantia nigra was found in the MCAO/R rats. EA intervention reduces BC in the contralateral substantia nigra in poststroke animals.

The DC is a graph theory metric that measures how many edges connected to a node and reflects the node's ability to connect directly with other nodes, having a neurobiological basis (Kwon et al., 2019). A high DC value demonstrated a large number of connections between a given node to others. In this study, LSD post hoc test revealed that a decreased DC value in the left somatosensory cortex was found in rats undergoing MCAO/R injury. This is consistent with clinical study showing that about 60% of stroke survivors have somatosensory deficits (Kessner et al., 2019). Furthermore, the anatomical structures with a changed DC referred to the left AcbSh in our study (Liu et al., 2019). In the basal ganglia, the nucleus accumbens is the primary input structure and can receive a large number of afferent signals from the limbic cortex to regulate goal‐directed behavior (Benoit‐Marand & O'Donnell, 2008). Previously, Vahid‐Ansari et al. established that endothelin‐1‐induced cerebral ischemia in male C57BL6 mice leads to chronic anxiety and depressive phenotypes. Stroke activated the glutamate cells of the nucleus accumbens (Vahid‐Ansari & Albert, 2018). Widmer et al. (2019) indicated that the nucleus accumbens, which is a component of the ventral striatum, was undamaged in all participants, but stroke patients showed significantly hypoactivation, which may be an indirect effect of stroke on activation patterns rather than direct damage to this area. The effect of EA intervention may be associated with the inhibition of AcbSh overactivation.

Neurological deficits caused by stroke are common among adults and often lead to functional impairments (Li et al., 2023). Higher scores indicate an infarction site located in a functionally important brain region or a larger lesion, which results in poor prognosis due to serious neurological deficits (Lu et al., 2022). Neurological function recovery is enormous importance to stroke patients because the deterioration of neurological function affects their cognitive, sensory, and motor functions directly (Jin et al., 2022; Wang et al., 2018). In this study, our results indicated that EA may be a promising treatment for improving neurological deficits after ischemic stroke. Combined with the results of imaging, EA could promote the activation of hippocampal, reduce BC in the right substantia nigra, and inhibit the overactivation of left AcbSh. Therefore, cognitive and motor‐associated brain areas participate in the EA mechanism of ischemic stroke, implying that EA may facilitate neurological recovery by modulating these brain regions.

There are some limitations to this study. First, a relatively small sample size may reduce the confidence of these results, and there were not significant when corrected for multiple comparisons. It is necessary to expand the sample size in the following experiment. Second, we only selected one time point to study rather than longitudinal observations. Further research should also consider conducting observations at multiple time points after surgery. Furthermore, MCAO/R is a classical model of cerebral ischemia, and individual rat vascular variation increases the uncertainty factor for the successful establishment of MCAO/R model. Finally, we did not set up MCAO/R + sham EA group, and future studies could observe changes of topological organization of functional networks within the brain in MCAO/R + sham EA group.

## 5 CONCLUSION

In conclusion, graph theory studies of functional networks show significant changes in the brain regions associated with the process of cognitive and motor functions. These findings provide an effective supplement for the neural mechanism of ischemic stroke. There has been a significant change in the topological architecture of the functional brain networks after EA at ST36 and LI11, and these changes are associated with acupoint specificity, so it can provide partial evidence for the brain function regulation related to acupuncture acupoint stimulation.

## AUTHOR CONTRIBUTIONS

**Si‐Si Li**: Conceptualization; data curation; validation; writing—original draft. **Xiang‐Xin Xing**: Formal analysis; visualization; writing—original draft. **Xu‐Yun Hua**: Funding acquisition; supervision; writing—original draft. **Yu‐Wen Zhang**: Data curation. **Jia‐Jia Wu**: Formal analysis; funding acquisition; visualization. **Chun‐Lei Shan**: Supervision. **He Wang**: Conceptualization; resources; writing—review and editing. **Mou‐Xiong Zheng**: Conceptualization; funding acquisition; validation; writing—review and editing. **Jian‐Guang Xu**: Conceptualization; funding acquisition; writing—review and editing.

## CONFLICT OF INTEREST STATEMENT

The authors declare no conflicts of interest.

### PEER REVIEW

The peer review history for this article is available at https://publons.com/publon/10.1002/brb3.3504.

## Data Availability

The datasets used and analyzed in the current study are available from the corresponding author on reasonable request.

## References

[brb33504-bib-0001] Aihara, T. , Shimokawa, T. , Ogawa, T. , Okada, Y. , Ishikawa, A. , Inoue, Y. , & Yamashita, O. (2020). Resting‐state functional connectivity estimated with hierarchical Bayesian diffuse optical tomography. Frontiers in Neuroscience, 14, 32. 32082110 10.3389/fnins.2020.00032 PMC7005139

[brb33504-bib-0002] Benoit‐Marand, M. , & O'Donnell, P. (2008). D2 dopamine modulation of cortico‐accumbens synaptic responses changes during adolescence. European Journal of Neuroscience, 27, 1364–1372. 18331340 10.1111/j.1460-9568.2008.06107.x PMC2613327

[brb33504-bib-0003] Broome, K. , Hudson, I. , Potter, K. , Kulk, J. , Dunn, A. , Arm, J. , Zeffiro, T. , Cooper, G. , Tian, H. , & van Vliet, P. (2019). A modified reach‐to‐grasp task in a supine position shows coordination between elbow and hand movements after stroke. Frontiers in Neurology, 10, 408. 31139132 10.3389/fneur.2019.00408 PMC6518444

[brb33504-bib-0004] Cai, R. , Shen, G. , Wang, H. , & Guan, Y. (2018). Brain functional connectivity network studies of acupuncture: A systematic review on resting‐state fMRI. Journal of Integrative Medicine, 16, 26–33. 29397089 10.1016/j.joim.2017.12.002

[brb33504-bib-0005] Chavez, L. M. , Huang, S. S. , MacDonald, I. , Lin, J. G. , Lee, Y. C. , & Chen, Y. H. (2017). Mechanisms of acupuncture therapy in ischemic stroke rehabilitation: A literature review of basic studies. International Journal of Molecular Sciences, 18, 2270. 29143805 10.3390/ijms18112270 PMC5713240

[brb33504-bib-0006] Chen, H. S. , Qi, S. H. , & Shen, J. G. (2017). One‐compound‐multi‐target: Combination prospect of natural compounds with thrombolytic therapy in acute ischemic stroke. Current Neuropharmacology, 15, 134–156. 27334020 10.2174/1570159X14666160620102055 PMC5327453

[brb33504-bib-0007] Chen, L. , Fan, X. , Li, H. , Ye, C. , Yu, H. , Gong, H. , Zeng, X. , Peng, D. , & Yan, L. (2018). Topological reorganization of the default mode network in severe male obstructive sleep apnea. Frontiers in Neurology, 9, 363. 29951028 10.3389/fneur.2018.00363 PMC6008385

[brb33504-bib-0008] Chen, X. , Liu, M. , Wu, Z. , & Cheng, H. (2020). Topological abnormalities of functional brain network in early‐stage Parkinson's disease patients with mild cognitive impairment. Frontiers in Neuroscience, 14, 616872. 33424546 10.3389/fnins.2020.616872 PMC7793724

[brb33504-bib-0009] Cheng, M. Y. , Aswendt, M. , & Steinberg, G. K. (2016). Optogenetic approaches to target specific neural circuits in post‐stroke recovery. Neurotherapeutics, 13, 325–340. 26701667 10.1007/s13311-015-0411-5 PMC4824024

[brb33504-bib-0010] Choi, M. J. , Kim, H. , Nah, H. W. , & Kang, D. W. (2019). Digital therapeutics: Emerging new therapy for neurologic deficits after stroke. Journal of Stroke, 21, 242–258. 31587534 10.5853/jos.2019.01963 PMC6780014

[brb33504-bib-0011] Coelho, A. , Fernandes, H. M. , Magalhaes, R. , Moreira, P. S. , Marques, P. , Soares, J. M. , Amorim, L. , Portugal‐Nunes, C. , Castanho, T. , Santos, N. C. , & Sousa, N. (2021). Reorganization of brain structural networks in aging: A longitudinal study. Journal of Neuroscience Research, 99, 1354–1376. 33527512 10.1002/jnr.24795 PMC8248023

[brb33504-bib-0012] Coloigner, J. , Phlypo, R. , Coates, T. D. , Lepore, N. , & Wood, J. C. (2017). Graph lasso‐based test for evaluating functional brain connectivity in sickle cell disease. Brain Connectivity, 7, 443–453. 28747064 10.1089/brain.2016.0474 PMC5647492

[brb33504-bib-0013] Fan, D. , Rich, D. , Holtzman, T. , Ruther, P. , Dalley, J. W. , Lopez, A. , Rossi, M. A. , Barter, J. W. , Salas‐Meza, D. , Herwik, S. , Holzhammer, T. , Morizio, J. , & Yin, H. H. (2011). A wireless multi‐channel recording system for freely behaving mice and rats. PLoS ONE, 6, e22033. 21765934 10.1371/journal.pone.0022033 PMC3134473

[brb33504-bib-0014] Guo, J. , Niu, K. , Ma, B. F. , Sun, L. N. , Fang, Q. W. , & An, J. X. (2023). Electroacupuncture ameliorates surgery‐induced spatial memory deficits by promoting mitophagy in rats. Annals of Translational Medicine, 11, 74. 36819507 10.21037/atm-22-6262 PMC9929787

[brb33504-bib-0015] Han, J. S. (2004). Acupuncture and endorphins. Neuroscience Letters, 361, 258–261. 15135942 10.1016/j.neulet.2003.12.019

[brb33504-bib-0016] Han, M. , Cui, J. , Xiao, Y. , Xiao, D. , Jiao, J. , Peng, Q. , Tian, F. , Tang, X. , Zhang, J. , & Jiang, Q. (2020). Acupuncture for primary fibromyalgia: Study protocol of a randomized controlled trial. Trials, 21, 538. 32552731 10.1186/s13063-020-04317-y PMC7301472

[brb33504-bib-0017] Han, X. , Jin, H. , Li, K. , Ning, Y. , Jiang, L. , Chen, P. , Liu, H. , Zhang, Y. , Zhang, H. , Tan, Z. , Cui, F. , Ren, Y. , Bai, L. , & Zou, Y. (2020). Acupuncture modulates disrupted whole‐brain network after ischemic stroke: Evidence based on graph theory analysis. Neural Plasticity, 2020, 8838498. 32922447 10.1155/2020/8838498 PMC7453235

[brb33504-bib-0018] Heo, J. H. , Nam, H. S. , Kim, Y. D. , Choi, J. K. , Kim, B. M. , Kim, D. J. , & Kwon, I. (2020). Pathophysiologic and therapeutic perspectives based on thrombus histology in stroke. Journal of Stroke, 22, 64–75. 32027792 10.5853/jos.2019.03440 PMC7005358

[brb33504-bib-0019] Hu, J. , Li, C. , Hua, Y. , Liu, P. , Gao, B. , Wang, Y. , & Bai, Y. (2020). Constraint‐induced movement therapy improves functional recovery after ischemic stroke and its impacts on synaptic plasticity in sensorimotor cortex and hippocampus. Brain Research Bulletin, 160, 8–23. 32298779 10.1016/j.brainresbull.2020.04.006

[brb33504-bib-0020] Huang, S. , Huang, D. , Zhao, J. , & Chen, L. (2017). Electroacupuncture promotes axonal regeneration in rats with focal cerebral ischemia through the downregulation of Nogo‐A/NgR/RhoA/ROCK signaling. Experimental and Therapeutic Medicine, 14, 905–912. 28810542 10.3892/etm.2017.4621 PMC5526169

[brb33504-bib-0021] Jin, Z. , Guo, F. , & Li, Y. (2022). Effects of systemic rehabilitation nursing combined with WeChat publicity and education on the early cognitive function and living quality of the patients with cerebral arterial thrombosis. Journal of Healthcare Engineering, 2022, 7396950. 35251575 10.1155/2022/7396950 PMC8894030

[brb33504-bib-0022] Kessner, S. S. , Schlemm, E. , Cheng, B. , Bingel, U. , Fiehler, J. , Gerloff, C. , & Thomalla, G. (2019). Somatosensory deficits after ischemic stroke. Stroke; A Journal of Cerebral Circulation, 50, 1116–1123. 10.1161/STROKEAHA.118.023750 30943883

[brb33504-bib-0023] Kim, Y. I. , Kim, S. S. , Sin, R. S. , Pu, Y. J. , Ri, G. , & Rim, K. S. (2018). Study on the cerebral blood flow regulatory features of acupuncture at acupoints of the governor vessel. Medical Acupuncture, 30, 192–197. 30147820 10.1089/acu.2018.1285 PMC6106755

[brb33504-bib-0024] Kwon, H. , Choi, Y. H. , & Lee, J. M. (2019). A *physarum* centrality measure of the human brain network. Scientific Reports, 9, 5907. 30976010 10.1038/s41598-019-42322-7 PMC6459855

[brb33504-bib-0025] Lai, X. , Zhang, J. , Chen, J. , Lai, C. , & Huang, C. (2020). Is electroacupuncture safe and effective for treatment of stress urinary incontinence in women? A systematic review and meta‐analysis. The Journal of International Medical Research, 48, 300060520948337. 33045874 10.1177/0300060520948337 PMC7570783

[brb33504-bib-0026] Latora, V. , & Marchiori, M. (2001). Efficient behavior of small‐world networks. Physical Review Letter, 87, 198701. 10.1103/PhysRevLett.87.198701 11690461

[brb33504-bib-0027] Lee, J. D. , Chon, J. S. , Jeong, H. K. , Kim, H. J. , Yun, M. , Kim, D. Y. , Kim, D. I. , Park, C. I. , & Yoo, H. S. (2003). The cerebrovascular response to traditional acupuncture after stroke. Neuroradiology, 45, 780–784. 12942221 10.1007/s00234-003-1080-3

[brb33504-bib-0028] Lessard, A. , & Couture, R. (2001). Modulation of cardiac activity by tachykinins in the rat substantia nigra. British Journal of Pharmacology, 134, 1749–1759. 11739252 10.1038/sj.bjp.0704401 PMC1572893

[brb33504-bib-0029] Li, B. , Xi, W. , Bai, Y. , Liu, X. , Zhang, Y. , Li, L. , Bian, L. , Liu, C. , Tang, Y. , Shen, L. , Yang, L. , Gu, X. , Xie, J. , Zhou, Z. , Wang, Y. , Yu, X. , Wang, J. , Chao, J. , Han, B. , & Yao, H. (2023). FTO‐dependent mA modification of Plpp3 in circSCMH1‐regulated vascular repair and functional recovery following stroke. Nature Communications, 14, 489. 10.1038/s41467-023-36008-y PMC9886939 36717587

[brb33504-bib-0030] Li, S. , Hua, X. , Zheng, M. , Wu, J. , Ma, Z. , Xing, X. , Ma, J. , Shan, C. , & Xu, J. (2022). Electroacupuncture treatment improves motor function and neurological outcomes after cerebral ischemia/reperfusion injury. Neural Regeneration Research, 17, 1545–1555. 34916440 10.4103/1673-5374.330617 PMC8771092

[brb33504-bib-0031] Li, S. , Hua, X. , Zheng, M. , Wu, J. , Ma, Z. , Xing, X. , Ma, J. , Zhang, J. , Shan, C. , & Xu, J. (2021). PLXNA2 knockdown promotes M2 microglia polarization through mTOR/STAT3 signaling to improve functional recovery in rats after cerebral ischemia/reperfusion injury. Experimental Neurology, 346, 113854. 34474008 10.1016/j.expneurol.2021.113854

[brb33504-bib-0032] Li, X. , Cai, L. , Jiang, X. , Liu, X. , Wang, J. , Yang, T. , & Wang, F. (2021). Resting‐state fMRI in studies of acupuncture. Evidence‐Based Complementary and Alternative Medicine, 2021, 6616060. 33859708 10.1155/2021/6616060 PMC8009717

[brb33504-bib-0033] Liao, X. , Vasilakos, A. V. , & He, Y. (2017). Small‐world human brain networks: Perspectives and challenges. Neuroscience and Biobehavioral Reviews, 77, 286–300. 28389343 10.1016/j.neubiorev.2017.03.018

[brb33504-bib-0034] Liu, B. , Chen, J. , Wang, J. , Liu, X. , Duan, X. , Shang, X. , Long, Y. , Chen, Z. , Li, X. , Huang, Y. , & He, Y. (2012). Altered small‐world efficiency of brain functional networks in acupuncture at ST36: A functional MRI study. PLoS ONE, 7, e39342. 22761766 10.1371/journal.pone.0039342 PMC3382219

[brb33504-bib-0035] Liu, W. , Wang, X. , Yang, S. , Huang, J. , Xue, X. , Zheng, Y. , Shang, G. , Tao, J. , & Chen, L. (2016). Electroacupunctre improves motor impairment via inhibition of microglia‐mediated neuroinflammation in the sensorimotor cortex after ischemic stroke. Life Sciences, 151, 313–322. 26979777 10.1016/j.lfs.2016.01.045

[brb33504-bib-0036] Liu, W. F. , Shu, Y. Q. , Zhu, P. W. , Li, B. , Shi, W. Q. , Lin, Q. , Liu, Y. X. , Zhang, M. Y. , Min, Y. L. , Yuan, Q. , & Shao, Y. (2019). The cerebellum posterior lobe associates with the exophthalmos of primary hyperthyroidism: A resting‐state fMRI study. International Journal of Endocrinology, 2019, 8135671. 31885561 10.1155/2019/8135671 PMC6914989

[brb33504-bib-0037] Liu, X. , Yan, Z. , Wang, T. , Yang, X. , Feng, F. , Fan, L. , & Jiang, J. (2015). Connectivity pattern differences bilaterally in the cerebellum posterior lobe in healthy subjects after normal sleep and sleep deprivation: A resting‐state functional MRI study. Neuropsychiatric Disease and Treatment, 11, 1279–1289. 26064046 10.2147/NDT.S84204 PMC4451850

[brb33504-bib-0038] Longa, E. Z. , Weinstein, P. R. , Carlson, S. , & Cummins, R. (1989). Reversible middle cerebral artery occlusion without craniectomy in rats. Stroke; A Journal of Cerebral Circulation, 20, 84–91. 10.1161/01.str.20.1.84 2643202

[brb33504-bib-0039] Lu, D. , Qu, C. , Goussev, A. , Jiang, H. , Lu, C. , Schallert, T. , Mahmood, A. , Chen, J. , Li, Y. , & Chopp, M. (2007). Statins increase neurogenesis in the dentate gyrus, reduce delayed neuronal death in the hippocampal CA3 region, and improve spatial learning in rat after traumatic brain injury. Journal of Neurotrauma, 24, 1132–1146. 17610353 10.1089/neu.2007.0288 PMC1971229

[brb33504-bib-0040] Lu, Q. , Bai, Q. , Ren, H. , Zhu, B. , Jiang, T. , Peng, C. , & Chen, X. (2022). Effectiveness and predictors of poor prognosis following intravenous thrombolysis in patients with wake‐up ischemic stroke guided by rapid MRI. Neuropsychiatric Disease and Treatment, 18, 317–325. 35210778 10.2147/NDT.S351524 PMC8860628

[brb33504-bib-0041] Ma, J. , Kang, H. J. , Kim, J. Y. , Jeong, H. S. , Im, J. J. , Namgung, E. , Kim, M. J. , Lee, S. , Kim, T. D. , Oh, J. K. , Chung, Y. A. , Lyoo, I. K. , Lim, S. M. , & Yoon, S. (2017). Network attributes underlying intellectual giftedness in the developing brain. Scientific Reports, 7, 11321. 28900176 10.1038/s41598-017-11593-3 PMC5596014

[brb33504-bib-0042] Marzullo, A. , Kocevar, G. , Stamile, C. , Durand‐Dubief, F. , Terracina, G. , Calimeri, F. , & Sappey‐Marinier, D. (2019). Classification of multiple sclerosis clinical profiles via graph convolutional neural networks. Frontiers in Neuroscience, 13, 594. 31244599 10.3389/fnins.2019.00594 PMC6581753

[brb33504-bib-0043] Moreno, M. , Minjarez, C. , Vigil, J. , Orfila, J. , Schmidt, R. , Burch, A. , Carter, D. , Kubesh, M. , Yonchek, J. , Dietz, R. , & Quillinan, N. (2022). Differences in hippocampal plasticity and memory outcomes in anterior versus posterior cerebellar stroke. Neurobiology of Disease, 168, 105701. 35337949 10.1016/j.nbd.2022.105701 PMC9047011

[brb33504-bib-0044] Murphy, T. H. , & Corbett, D. (2009). Plasticity during stroke recovery: From synapse to behaviour. Nature Reviews Neuroscience, 10, 861–872. 19888284 10.1038/nrn2735

[brb33504-bib-0045] Nakane, M. , Tamura, A. , Nagaoka, T. , & Hirakawa, K. (1997). MR detection of secondary changes remote from ischemia: Preliminary observations after occlusion of the middle cerebral artery in rats. AJNR, American Journal of Neuroradiology, 18, 945–950. 9159375 PMC8338101

[brb33504-bib-0046] Ohe, Y. , Uchino, A. , Horiuchi, Y. , Maruyama, H. , Deguchi, I. , Fukuoka, T. , Kato, Y. , Nagoya, H. , Dembo, T. , & Tanahashi, N. (2013). Magnetic resonance imaging investigation of secondary degeneration of the mesencephalic substantia nigra after cerebral infarction. Journal of Stroke and Cerebrovascular Diseases, 22, 58–65. 21784662 10.1016/j.jstrokecerebrovasdis.2011.06.006

[brb33504-bib-0047] Pang, R. , Wang, D. , Chen, T. S. R. , Yang, A. , Yi, L. , Chen, S. , Wang, J. , Wu, K. , Zhao, C. , Liu, H. , Ai, Y. , Yang, A. , & Sun, J. (2022). Reorganization of prefrontal network in stroke patients with dyskinesias: Evidence from resting‐state functional near‐infrared spectroscopy. Journal of Biophotonics, 15, e202200014. 35324088 10.1002/jbio.202200014

[brb33504-bib-0048] Sanchez‐Bezanilla, S. , Aberg, N. D. , Crock, P. , Walker, F. R. , Nilsson, M. , Isgaard, J. , & Ong, L. K. (2020). Growth hormone treatment promotes remote hippocampal plasticity after experimental cortical stroke. International Journal of Molecular Sciences, 21, 4563. 32604953 10.3390/ijms21124563 PMC7349868

[brb33504-bib-0049] Schwarz, A. J. , Danckaert, A. , Reese, T. , Gozzi, A. , Paxinos, G. , Watson, C. , Merlo‐Pich, E. V. , & Bifone, A. (2006). A stereotaxic MRI template set for the rat brain with tissue class distribution maps and co‐registered anatomical atlas: Application to pharmacological MRI. Neuroimage, 32, 538–550. 16784876 10.1016/j.neuroimage.2006.04.214

[brb33504-bib-0050] Shetty, M. S. , Sharma, M. , & Sajikumar, S. (2017). Chelation of hippocampal zinc enhances long‐term potentiation and synaptic tagging/capture in CA1 pyramidal neurons of aged rats: Implications to aging and memory. Aging Cell, 16, 136–148. 27633878 10.1111/acel.12537 PMC5242293

[brb33504-bib-0051] Shi, M. , Liu, S. , Chen, H. , Geng, W. , Yin, X. , Chen, Y. C. , & Wang, L. (2021). Disrupted brain functional network topology in unilateral acute brainstem ischemic stroke. Brain Imaging and Behavior, 15, 444–452. 32705464 10.1007/s11682-020-00353-z

[brb33504-bib-0052] Sporns, O. (2018). Graph theory methods: Applications in brain networks. Dialogues in Clinical Neuroscience, 20, 111–121. 30250388 10.31887/DCNS.2018.20.2/osporns PMC6136126

[brb33504-bib-0053] Tang, X. , Wang, C. , Xia, L. , Zhu, W. , Zhao, L. , & Zhu, W. (2012). Volumetric MRI and 1H MRS study of hippocampus in unilateral MCAO patients: Relationship between hippocampal secondary damage and cognitive disorder following stroke. European Journal of Radiology, 81, 2788–2793. 21945401 10.1016/j.ejrad.2011.08.010

[brb33504-bib-0054] Tao, Y. , Liu, B. , Zhang, X. , Li, J. , Qin, W. , Yu, C. , & Jiang, T. (2015). The structural connectivity pattern of the default mode network and its association with memory and anxiety. Frontiers in Neuroanatomy, 9, 152. 26635544 10.3389/fnana.2015.00152 PMC4659898

[brb33504-bib-0055] Toscano, M. , Celletti, C. , Vigano, A. , Altarocca, A. , Giuliani, G. , Jannini, T. B. , Mastria, G. , Ruggiero, M. , Maestrini, I. , Vicenzini, E. , Altieri, M. , Camerota, F. , & Di Piero, V. (2019). Short‐term effects of focal muscle vibration on motor recovery after acute stroke: A pilot randomized sham‐controlled study. Frontiers in Neurology, 10, 115. 30873102 10.3389/fneur.2019.00115 PMC6401608

[brb33504-bib-0056] Urits, I. , Patel, M. , Putz, M. E. , Monteferrante, N. R. , Nguyen, D. , An, D. , Cornett, E. M. , Hasoon, J. , Kaye, A. D. , & Viswanath, O. (2020). Acupuncture and its role in the treatment of migraine headaches. Neurology and Therapy, 9, 375–394. 33001385 10.1007/s40120-020-00216-1 PMC7606388

[brb33504-bib-0057] Vahid‐Ansari, F. , & Albert, P. R. (2018). Chronic fluoxetine induces activity changes in recovery from poststroke anxiety, depression, and cognitive impairment. Neurotherapeutics, 15, 200–215. 29204954 10.1007/s13311-017-0590-3 PMC5794702

[brb33504-bib-0058] Vecchio, F. , Pappalettera, C. , Miraglia, F. , Deinite, G. , Manenti, R. , Judica, E. , Caliandro, P. , & Rossini, P. M. (2023). Prognostic role of hemispherical functional connectivity in stroke: A study via graph theory versus coherence of electroencephalography rhythms. Stroke; A Journal of Cerebral Circulation, 54, 499–508. 10.1161/STROKEAHA.122.040747 36416129

[brb33504-bib-0059] Waki, H. , Suzuki, T. , Tanaka, Y. , Tamai, H. , Minakawa, Y. , Miyazaki, S. , Yoshida, N. , Uebaba, K. , Imai, K. , & Hisajima, T. (2017). Effects of electroacupuncture to the trigeminal nerve area on the autonomic nervous system and cerebral blood flow in the prefrontal cortex. Acupuncture in Medicine, 35, 339–344. 28765118 10.1136/acupmed-2016-011247

[brb33504-bib-0060] Wang, J. , Niu, J. F. , Li, G. H. , & Zhao, X. F. (2019). [Review of the mechanism of essential hypertension treated by acupuncture]. Zhongguo Zhen Jiu, 39, 224–228. 30942045 10.13703/j.0255-2930.2019.02.031

[brb33504-bib-0061] Wang, P. , Li, W. , Zhu, H. , Liu, X. , Yu, T. , Zhang, D. , & Zhang, Y. (2022). Reorganization of the brain structural covariance network in ischemic moyamoya disease revealed by graph theoretical analysis. Frontiers in Aging Neuroscience, 14, 788661. 35721027 10.3389/fnagi.2022.788661 PMC9201423

[brb33504-bib-0062] Wang, X. , Xuan, W. , Zhu, Z. , Li, Y. , Zhu, H. , Zhu, L. , Fu, D. , Yang, L. , Li, P. , & Yu, W. (2018). The evolving role of neuro‐immune interaction in brain repair after cerebral ischemic stroke. CNS Neuroscience & Therapeutics, 24, 1100–1114. 30350341 10.1111/cns.13077 PMC6489764

[brb33504-bib-0063] Wanni Arachchige, P. , Karunarathna, S. , Meidian, A. , Ueda, R. , Uchida, W. , Abo, M. , & Senoo, A. (2021). Structural connectivity changes in the motor execution network after stroke rehabilitation. Restorative Neurology and Neuroscience, 39, 237–245. 34275914 10.3233/RNN-211148 PMC8543268

[brb33504-bib-0064] Watson, C. G. , Stopp, C. , Newburger, J. W. , & Rivkin, M. J. (2018). Graph theory analysis of cortical thickness networks in adolescents with d‐transposition of the great arteries. Brain and Behavior, 8, e00834. 29484251 10.1002/brb3.834 PMC5822582

[brb33504-bib-0065] Widmer, M. , Lutz, K. , & Luft, A. R. (2019). Reduced striatal activation in response to rewarding motor performance feedback after stroke. NeuroImage: Clinical, 24, 102036. 31698315 10.1016/j.nicl.2019.102036 PMC6978223

[brb33504-bib-0066] Xiong, L. , Lu, Z. , Hou, L. , Zheng, H. , Zhu, Z. , Wang, Q. , & Chen, S. (2003). Pretreatment with repeated electroacupuncture attenuates transient focal cerebral ischemic injury in rats. Chinese Medical Journal, 116, 108–111. 12667400

[brb33504-bib-0067] Xu, J. (2015). Implications of cortical balanced excitation and inhibition, functional heterogeneity, and sparseness of neuronal activity in fMRI. Neuroscience and Biobehavioral Reviews, 57, 264–270. 26341939 10.1016/j.neubiorev.2015.08.018 PMC4623927

[brb33504-bib-0068] Xue, X. , Wu, J. J. , Huo, B. B. , Xing, X. X. , Ma, J. , Li, Y. L. , Zheng, M. X. , Hua, X. Y. , & Xu, J. G. (2022). Age‐related alterations of brain metabolic network based on [18F]FDG‐PET of rats. Aging (Albany NY), 14, 923–942. 35077393 10.18632/aging.203851 PMC8833125

[brb33504-bib-0069] Yang, G. , Zhu, J. , Zhan, G. , Fan, G. , Deng, L. , Tang, H. , Jiang, X. , Chen, B. , & Yang, C. (2022). Mesenchymal stem cell‐derived neuron‐like cell transplantation combined with electroacupuncture improves synaptic plasticity in rats with intracerebral hemorrhage via mTOR/p70S6K signaling. Stem Cells International, 2022, 6450527. 35211177 10.1155/2022/6450527 PMC8863490

[brb33504-bib-0070] Yin, L. , Tang, T. , Lin, Y. , Yang, M. , Liu, W. , & Liang, S. (2022). Functional connectivity of ipsilateral striatum in rats with ischemic stroke increased by electroacupuncture. Journal of Integrative Neuroscience, 21, 162. 36424737 10.31083/j.jin2106162

[brb33504-bib-0071] Zhang, J. , Li, Z. , Cao, X. , Zuo, L. , Wen, W. , Zhu, W. , Jiang, J. , Cheng, J. , Sachdev, P. , Liu, T. , & Wang, Y. (2020). Altered prefrontal‐basal ganglia effective connectivity in patients with poststroke cognitive impairment. Frontiers in Neurology, 11, 577482. 33391148 10.3389/fneur.2020.577482 PMC7772311

[brb33504-bib-0072] Zhang, J. , Liu, Y. , Li, Z. , Hu, Q. , Huang, X. , Lv, H. , Xu, J. , & Yu, H. (2023). Functional magnetic resonance imaging studies of acupuncture at ST36: A coordinate‐based meta‐analysis. Frontiers in Neuroscience, 17, 1180434. 37360179 10.3389/fnins.2023.1180434 PMC10287969

[brb33504-bib-0073] Zhang, J. , Lu, C. , Wu, X. , Nie, D. , & Yu, H. (2021). Neuroplasticity of acupuncture for stroke: An evidence‐based review of MRI. Neural Plasticity, 2021, 2662585. 34456996 10.1155/2021/2662585 PMC8397547

[brb33504-bib-0074] Zhang, K. , Zhang, Q. , Deng, J. , Li, J. , Li, J. , Wen, L. , Ma, J. , & Li, C. (2019). ALK5 signaling pathway mediates neurogenesis and functional recovery after cerebral ischemia/reperfusion in rats via Gadd45b. Cell Death & Disease, 10, 360. 31043581 10.1038/s41419-019-1596-z PMC6494915

[brb33504-bib-0076] Zhu, Y. , Bai, L. , Liang, P. , Kang, S. , Gao, H. , & Yang, H. (2017). Disrupted brain connectivity networks in acute ischemic stroke patients. Brain Imaging & Behavior, 11, 444–453. 26883758 10.1007/s11682-016-9525-6

[brb33504-bib-0077] Zou, F. , Lin, Y. F. , Chen, S. G. , Cao, L. , Wang, H. R. , Ye, B. , Wang, Q. , Jie‐Ying, H. , & Jia, J. (2020). The impact of electroacupuncture at hegu, shousanli, and quchi based on the theory “treating flaccid paralysis by Yangming alone” on stroke patients' EEG: A pilot study. Evidence‐Based Complementary and Alternative Medicine, 2020, 8839491. 33299460 10.1155/2020/8839491 PMC7707989

